# 2-({3-[(2*R*,4*S*,5*R*)-4-Hydr­oxy-5-hydroxy­methyl-2,3,4,5-tetra­hydro­furan-2-yl]-5-methyl-2,6-dioxo-1,2,3,6-tetra­hydro­pyrimidin-1-yl}meth­yl)isoindoline-1,3-dione

**DOI:** 10.1107/S1600536809003365

**Published:** 2009-01-31

**Authors:** Mark Daniel Bartholomä, Wayne Ouellette, Jon Zubieta

**Affiliations:** a Department of Chemistry, Syracuse University, New York 13244, USA; b Department of Chemistry, Syracuse University, New York 13244, USA

## Abstract

The title compound, C_19_H_19_N_3_O_7_, is a thymidine derivative and serves as an inter­mediate in the synthesis of a ^99*m*^Tc radiolabeled nucleoside analog. Inter­molecular O—H⋯O hydrogen bonding is observed between the hydr­oxy functionalities of the ribose unit themselves as well as between a hydr­oxy group and an O atom of the phthalimide group of an adjacent mol­ecule. The mol­ecules are stacked on top of each other in the direction of the *a* axis. The crystal packing is further stabilized by weak intra- and inter­molecular C—H⋯O hydrogen bonds. The absolute configuration of the compound is known from the synthesis.

## Related literature

For general background on human thymidine kinase 1 (hTK-1), see: Arner & Eriksson (1995 ▶); Bello (1974 ▶); Eriksson *et al.* (2002 ▶). For related literature, see: Wei *et al.* (2005 ▶); Bartholomä *et al.* (2009 ▶); Flack (1983 ▶). For crystal structure of hTK-1, see: Welin *et al.* (2004 ▶).
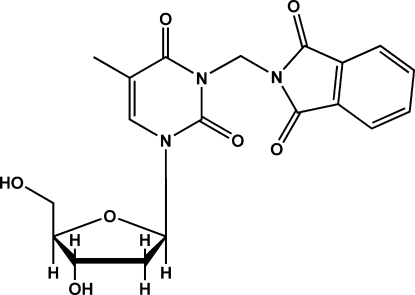


## Experimental

### 

#### Crystal data

C_19_H_19_N_3_O_7_
*M* *_r_* = 401.37
Monoclinic, 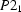

*a* = 4.9334 (4) Å
*b* = 11.6287 (9) Å
*c* = 15.3208 (12) Å
β = 91.430 (2)°
*V* = 878.67 (12) Å^3^
*Z* = 2
Mo *K*α radiation
μ = 0.12 mm^−1^
*T* = 90 (2) K
0.28 × 0.22 × 0.08 mm

#### Data collection

Bruker SMART APEX diffractometer
Absorption correction: multi-scan (*SADABS*; Bruker, 1998 ▶) *T*_min_ = 0.968, *T*_max_ = 0.991
9291 measured reflections
4344 independent reflections
3949 reflections with *I* > 2σ(*I*)
*R*_int_ = 0.028

#### Refinement

*R*[*F* ^2^ > 2σ(*F* ^2^)] = 0.045
*wR*(*F* ^2^) = 0.097
*S* = 1.08
4344 reflections
269 parameters
1 restraint
H atoms treated by a mixture of independent and constrained refinement
Δρ_max_ = 0.30 e Å^−3^
Δρ_min_ = −0.21 e Å^−3^

### 

Data collection: *SMART* (Bruker, 1998 ▶); cell refinement: *SAINT* (Bruker, 1998 ▶); data reduction: *SAINT*; program(s) used to solve structure: *SHELXS97* (Sheldrick, 2008 ▶); program(s) used to refine structure: *SHELXL97* (Sheldrick, 2008 ▶); molecular graphics: *DIAMOND* (Brandenburg & Putz, 1999 ▶); software used to prepare material for publication: *SHELXTL* (Sheldrick, 2008 ▶).

## Supplementary Material

Crystal structure: contains datablocks I, global. DOI: 10.1107/S1600536809003365/fb2127sup1.cif

Structure factors: contains datablocks I. DOI: 10.1107/S1600536809003365/fb2127Isup2.hkl

Additional supplementary materials:  crystallographic information; 3D view; checkCIF report

## Figures and Tables

**Table 1:** Hydrogen-bond geometry (Å, °) *Cg*1 and *Cg*2 are the centroids of the N1/C6/C7/C9/N2/C10 and N3/C12/C13/C18/C19 rings, respectively.

*D*—H⋯*A*	*D*—H	H⋯*A*	*D*⋯*A*	*D*—H⋯*A*
O1—H1⋯O2^i^	0.81 (3)	2.02 (3)	2.815 (2)	170 (3)
O2—H2*A*⋯O6^i^	0.84 (3)	1.92 (3)	2.698 (2)	155 (3)
C3—H3⋯O6^ii^	1.00	2.45	3.445 (2)	171
C8—H8*A*⋯O7^iii^	0.98	2.43	3.341 (3)	154
C14—H14⋯O5^iv^	0.95	2.44	3.116 (3)	128
C15—H15⋯O7^iv^	0.95	2.60	3.484 (3)	155
C5—H5⋯O4	1.00	2.33	2.740 (2)	104
C11—H11*A*⋯O4	0.99	2.29	2.747 (2)	107
C11—H11*B*⋯O7	0.99	2.52	2.915 (3)	104
C8—H8*B*⋯*Cg*1^v^	0.98	2.71	3.534 (2)	143
C11—H11*A*⋯*Cg*2^vi^	0.99	2.73	3.611 (2)	149

Symmetry codes: (i) 
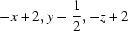
; (ii) 
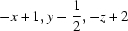
; (iii) 
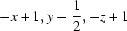
; (iv) 
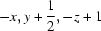
; (v) 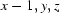; (vi) 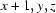.

**Fig. 1.**
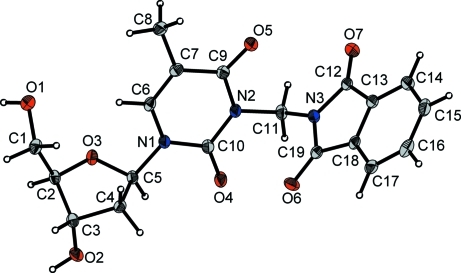
View of the title structure, with the atom numbering scheme and the displacement ellipsoids drawn at 50% probability level.

## supplementary crystallographic information

### Comment

In recent years, the development of radiolabeled nucleosides and nucleoside analogs has gained increased interest because of their potential use as probes for tumor cell proliferation. The targeted enzyme is the human cytosolic thymidine kinase (hTK-1), an enzyme of the pyrimidine salvage pathway, which catalyzes the phosphorylation of thymidine and uridine as natural substrates to their corresponding 5'-monophosphates (Welin *et al.*, 2004). These monophosphates are precursors of the DNA synthesis. Further conversion to the di- and triphosphates by nucleoside mono- and diphosphate kinases finally results in DNA incorporation. Most important, proliferating cells such as tumor cells show a dramatically increased hTK-1 activity compared to quiescent cells which makes hTK-1 an attractive target for selective imaging and therapeutic applications (Bello, 1974). Moreover, nucleosides are taken up by proliferating cells through facilitated diffusion but the cellular efflux of the corresponding negatively charged 5'-monophosphates is retarded (Arner & Eriksson, 1995). Thus, a radiolabeled nucleoside analog would be trapped inside the cell resulting in an accumulation in tissue with elevated hTK-1 activity such as tumor cells. The main challenge is the development of a nucleoside derivative which retains its substrate activity since hTK-1 has a narrow substrate specifity (Eriksson *et al.*, 2002). The literature on the interaction of thymidine derivatives with hTK-1 is not totally unambiguous about the effects of various substitutions. In general, major modifications of thymidine or uridine, respectively, led to a highly decreased activity of the corresponding compound. On the other hand, several derivatives modified either at the ribose and the base site are reported which retain their activity. To shed light on the effects of various modifications on the substrate activity, we prepared a library of nucleoside analogs that had been modified at different positions of the ribose and base moiety. With this library, we expand our SAAC concept (single amino acid chelate) on nucleosides for radioimaging and radiotherapeutic purposes (Wei *et al.* 2005, Bartholomä *et al.*, 2009).

2-((2,3-dihydro-3-((2*R*,4*S*,5*R*)-tetrahydro-4-hydroxy-5- (hydroxymethyl)furan-2-yl)-5-methyl-2,6-dioxopyrimidin-1(6*H*)-yl) methyl)isoindoline-1,3-dione is an intermediate in the synthesis of a ^99^*^m^*Tc radiolabeled nucleoside analog. The corresponding final product is a representative of the N-3 derivatized thymidine analogs with the shortest tether length applicable between the bioactive molecule and the chelate. The entire synthetic pathway will be described elsewhere. The SAAC chelate enables a chemically robust and inert coordination of the [*M*(CO)_3_]^+^ core (*M* = ^186^Re, ^188^Re, ^99^*^m^*Tc). ^99^*^m^*Tc with its ideal decay properties, low cost and good availability can be used for imaging purposes while the corresponding rhenium complexes would act as therapeutic counterparts.

2-((2,3-dihydro-3-((2*R*,4*S*,5*R*)-tetrahydro-4-hydroxy-5- (hydroxymethyl)furan-2-yl)-5-methyl-2,6-dioxopyrimidin-1(6*H*)-yl) methyl)isoindoline-1,3-dione shows strong intermolecular hydrogen bonding interactions of the type O—H···O as well as weak intra- and intermolecular C—H···O hydrogen bonds (Tab. 1). The O—H···O hydrogen bonding interaction occurs between the hydroxyl group at the 5' position of the sugar moiety (O1—H1C) and the oxygen atom O2 at the 2' position of an adjacent molecule (O2) with a O1—H1C···O2 distance of 2.815 (2) Å (Tab. 1). Another O—H···O hydrogen bonding interaction is observed between the hydroxy group at the 2' position of the ribose moiety (O2—H2A) and the oxygen atom of the phthalimide residue (O6) of the adjacent molecule. The corresponding O2—H2A···O6 distance is 2.698 (2) Å. The molecules are stacked on top of each other in direction of the *a* axis. There are π-π electron interactions between the aromatic rings N3\C12\C13\C18\C19 and C13\C14\C15\C16\C17\C18 of the phthalimide moiety. The distance between the centroids of these rings is 3.7245 (12) Å. Moreover, there are also C-H···π-electron ring interactions (Tab. 1). The ribose moiety of the nucleoside analog adopts a twist conformation with C2, O3, and C5 in plane. The atoms C3 and C4 are out of plane with d = 0.2356 (25) Å and d = -0.4099 (25) Å, respectively. The distance between C6 and C7 with 1.334 (3) Å is representative for the double bond character. The phthalimide residue has essentially a planar geometry. The absolute configuration of the compound is known by synthesis. All the bond lengths and angles are in expected ranges.

### Experimental

1 g (4.13 mmol) of 1-(2-deoxy-β-*D*-ribofuranosyl)thymine, 1.862 g (4.54 mmol) of phthalimidomethylpyridinium *p*-toluenesulfonate and 1.141 g (8.26 mmol) of K_2_CO_3_ were suspended in anhydrous *N*,*N*-dimethylformamide and kept at 50°C for 2 d. The solvent was removed in vacuum. 50 ml of water were added to the yellowish suspension and the aqueous phase was extracted twice with dichloromethane. The combined organic layers were washed with water and brine, dried with MgSO_4_ and evaporated to dryness. The crude product was purified by silica gel column chromatography using CH_2_Cl_2_:MeOH 15:1. The last fraction contained the product. Single crystals suitable for X-ray diffraction were obtained by recrystallizing the product in methanol yielding colorless plates. ^1^H NMR (d_4_-MeOD): *δ* = 1.90 (s, 3 H), 2.35 (m, 2 H), 3.87 (m, 2 H), 3.98 (dd, J = 3.20 Hz, 3.30 Hz, 1 H), 3.56 (dd, J = 4.41 Hz, J = 9.18 Hz, 1 H), 5.90 (s, 2 H), 6.24 (t, J = 6.53 Hz, 1 H), 7.52 (s, 1 H), 7.72 (m, 2 H), 7.84 (m, 2 H). p.p.m.. IR: *ν* = 3088, 2963, 2542, 1789, 1728, 1703, 1646, 1466, 1428, 1406, 1347, 1272, 1248, 1218, 1178, 1118, 1070, 1037, 1014, 992, 935, 892, 847, 769, 729, 716, 632, 608, 567, 532, 515, 491 cm^-1^.

### Refinement

All the H atoms were discernible in the difference electron density map. However, with exception of the hydroxyl hydrogens whose coordinates were refined freely they were situated into the idealized positions and refined by the riding model. The applied constraints: *C*—H_aryl_ = 0.95; *C*—H_methine_ = 1.00; *C*—H_methylene_ = 0.99 and *C*—H_methyl_ = 0.98Å, respectively, and included in the riding-model approximation with *U*_iso_(H) = 1.2*U*_eq_(C) or 1.5*U*_eq_(C/O) for methyl and hydroxyl H atoms. The Friedel pairs were not merged and the Flack absolute structure parameter converged to an indeterminate  value (Flack, 1983) with a large standard uncertainty (0.3 (9)). The absolute structure has been derived by the known structure of the precursors used in the synthesis.

### Crystal data

**Table:**

C_19_H_19_N_3_O_7_	*F*(000) = 420
*M**_r_* = 401.37	*D*_x_ = 1.517 Mg m^−^^3^
Monoclinic, *P*2_1_	Mo *K*α radiation, λ = 0.71073 Å
Hall symbol: P2yb	Cell parameters from 2431 reflections
*a* = 4.9334 (4) Å	θ = 2.2–27.9°
*b* = 11.6287 (9) Å	µ = 0.12 mm^−^^1^
*c* = 15.3208 (12) Å	*T* = 90 K
β = 91.430 (2)°	Plate, colorless
*V* = 878.67 (12) Å^3^	0.28 × 0.22 × 0.08 mm
*Z* = 2	

### Data collection

**Table:**

Bruker SMART APEX diffractometer	4344 independent reflections
Radiation source: fine-focus sealed tube	3949 reflections with *I* > 2σ(*I*)
graphite	*R*_int_ = 0.028
Detector resolution: 512 pixels mm^-1^	θ_max_ = 28.3°, θ_min_ = 2.2°
φ and ω scans	*h* = −6→6
Absorption correction: multi-scan (*SADABS*; Bruker, 1998)	*k* = −15→15
*T*_min_ = 0.968, *T*_max_ = 0.991	*l* = −20→20
9291 measured reflections	

### Refinement

**Table:**

Refinement on *F*^2^	Primary atom site location: structure-invariant direct methods
Least-squares matrix: full	Secondary atom site location: difference Fourier map
*R*[*F*^2^ > 2σ(*F*^2^)] = 0.045	Hydrogen site location: difference Fourier map
*wR*(*F*^2^) = 0.097	H atoms treated by a mixture of independent and constrained refinement
*S* = 1.08	*w* = 1/[σ^2^(*F*_o_^2^) + (0.0453*P*)^2^ + 0.1384*P*] where *P* = (*F*_o_^2^ + 2*F*_c_^2^)/3
4344 reflections	(Δ/σ)_max_ < 0.001
269 parameters	Δρ_max_ = 0.30 e Å^−^^3^
1 restraint	Δρ_min_ = −0.20 e Å^−^^3^
69 constraints	

### Special details

**Table:**

Geometry. All esds (except the esd in the dihedral angle between two l.s. planes) are estimated using the full covariance matrix. The cell esds are taken into account individually in the estimation of esds in distances, angles and torsion angles; correlations between esds in cell parameters are only used when they are defined by crystal symmetry. An approximate (isotropic) treatment of cell esds is used for estimating esds involving l.s. planes.
Refinement. Refinement of F^2^ against ALL reflections. The weighted R-factor wR and goodness of fit S are based on F^2^, conventional R-factors R are based on F, with F set to zero for negative F^2^. The threshold expression of F^2^ > 2sigma(F^2^) is used only for calculating R-factors(gt) etc. and is not relevant to the choice of reflections for refinement. R-factors based on F^2^ are statistically about twice as large as those based on F, and R- factors based on ALL data will be even larger.

### Fractional atomic coordinates and isotropic or equivalent isotropic displacement parameters (Å^2^)

**Table:**

	*x*	*y*	*z*	*U*_iso_*/*U*_eq_	
O1	0.6387 (3)	−0.27208 (14)	0.89260 (11)	0.0250 (3)	
H1	0.715 (6)	−0.331 (3)	0.9067 (19)	0.038*	
O2	1.1522 (3)	0.00883 (13)	1.06603 (10)	0.0207 (3)	
H2A	1.200 (5)	−0.027 (3)	1.1113 (18)	0.031*	
O3	0.9764 (3)	−0.07418 (12)	0.89077 (9)	0.0164 (3)	
O4	1.0136 (3)	0.24132 (13)	0.80115 (10)	0.0235 (3)	
O5	0.3376 (3)	0.16674 (13)	0.60019 (9)	0.0210 (3)	
O6	0.5554 (3)	0.43064 (14)	0.79515 (9)	0.0219 (3)	
O7	0.4438 (3)	0.39579 (13)	0.49918 (9)	0.0217 (3)	
N1	0.7609 (3)	0.07853 (14)	0.81636 (11)	0.0156 (3)	
N2	0.6792 (3)	0.20148 (14)	0.69845 (11)	0.0154 (3)	
N3	0.5461 (3)	0.39145 (15)	0.64784 (11)	0.0158 (3)	
C1	0.6506 (4)	−0.19797 (18)	0.96556 (14)	0.0188 (4)	
H1A	0.6331	−0.2443	1.0193	0.023*	
H1B	0.4948	−0.1444	0.9620	0.023*	
C2	0.9127 (4)	−0.12818 (16)	0.97232 (13)	0.0148 (4)	
H2	1.0654	−0.1799	0.9909	0.018*	
C3	0.8896 (4)	−0.03031 (18)	1.03767 (12)	0.0155 (4)	
H3	0.7713	−0.0503	1.0875	0.019*	
C4	0.7686 (4)	0.06349 (17)	0.97993 (13)	0.0151 (4)	
H4A	0.8044	0.1408	1.0048	0.018*	
H4B	0.5707	0.0531	0.9710	0.018*	
C5	0.9186 (4)	0.04568 (17)	0.89526 (13)	0.0150 (4)	
H5	1.0924	0.0899	0.8977	0.018*	
C6	0.5569 (4)	0.00709 (17)	0.78501 (13)	0.0159 (4)	
H6	0.5206	−0.0615	0.8163	0.019*	
C7	0.4083 (4)	0.02949 (17)	0.71310 (12)	0.0159 (4)	
C8	0.1942 (4)	−0.05012 (18)	0.67757 (13)	0.0193 (4)	
H8A	0.2571	−0.0860	0.6238	0.029*	
H8B	0.0277	−0.0066	0.6648	0.029*	
H8C	0.1576	−0.1099	0.7208	0.029*	
C9	0.4629 (4)	0.13441 (17)	0.66525 (13)	0.0154 (4)	
C10	0.8316 (4)	0.17875 (17)	0.77439 (13)	0.0162 (4)	
C11	0.7536 (4)	0.30277 (17)	0.64747 (14)	0.0171 (4)	
H11A	0.9254	0.3349	0.6717	0.021*	
H11B	0.7849	0.2791	0.5865	0.021*	
C12	0.4075 (4)	0.43033 (17)	0.57187 (13)	0.0165 (4)	
C13	0.2165 (4)	0.52024 (17)	0.60267 (13)	0.0167 (4)	
C14	0.0285 (4)	0.58521 (18)	0.55652 (14)	0.0198 (4)	
H14	0.0046	0.5772	0.4951	0.024*	
C15	−0.1251 (4)	0.66315 (19)	0.60343 (15)	0.0228 (5)	
H15	−0.2568	0.7091	0.5735	0.027*	
C16	−0.0891 (4)	0.67490 (19)	0.69306 (15)	0.0232 (5)	
H16	−0.1983	0.7279	0.7236	0.028*	
C17	0.1052 (4)	0.60998 (18)	0.73916 (15)	0.0200 (4)	
H17	0.1335	0.6191	0.8003	0.024*	
C18	0.2537 (4)	0.53243 (17)	0.69231 (13)	0.0167 (4)	
C19	0.4663 (4)	0.44926 (17)	0.72216 (13)	0.0166 (4)	

### Atomic displacement parameters (Å^2^)

**Table:**

	*U*^11^	*U*^22^	*U*^33^	*U*^12^	*U*^13^	*U*^23^
O1	0.0313 (9)	0.0176 (8)	0.0258 (8)	−0.0017 (7)	−0.0052 (7)	−0.0002 (7)
O2	0.0200 (7)	0.0219 (8)	0.0199 (7)	−0.0031 (6)	−0.0078 (6)	0.0045 (6)
O3	0.0187 (7)	0.0132 (7)	0.0175 (7)	0.0029 (6)	0.0042 (5)	0.0027 (6)
O4	0.0231 (8)	0.0181 (8)	0.0286 (8)	−0.0076 (6)	−0.0111 (6)	0.0083 (6)
O5	0.0237 (7)	0.0187 (8)	0.0201 (8)	−0.0032 (6)	−0.0056 (6)	0.0028 (6)
O6	0.0243 (7)	0.0254 (8)	0.0158 (7)	−0.0049 (6)	−0.0060 (6)	0.0019 (6)
O7	0.0280 (8)	0.0213 (8)	0.0157 (7)	−0.0004 (6)	−0.0007 (6)	0.0009 (6)
N1	0.0160 (8)	0.0140 (8)	0.0167 (8)	−0.0023 (7)	−0.0022 (6)	0.0033 (7)
N2	0.0160 (8)	0.0133 (8)	0.0169 (8)	−0.0031 (6)	−0.0014 (6)	0.0034 (6)
N3	0.0169 (8)	0.0144 (8)	0.0159 (8)	−0.0015 (6)	−0.0024 (6)	0.0028 (7)
C1	0.0178 (10)	0.0155 (10)	0.0232 (11)	−0.0024 (8)	0.0011 (8)	0.0024 (8)
C2	0.0125 (9)	0.0140 (10)	0.0179 (10)	0.0022 (7)	0.0001 (7)	0.0038 (7)
C3	0.0143 (9)	0.0161 (9)	0.0161 (9)	−0.0004 (7)	0.0022 (7)	0.0028 (8)
C4	0.0151 (8)	0.0121 (9)	0.0182 (9)	0.0005 (7)	−0.0011 (7)	−0.0009 (7)
C5	0.0147 (9)	0.0130 (9)	0.0171 (9)	−0.0001 (7)	−0.0021 (7)	0.0031 (7)
C6	0.0168 (9)	0.0127 (9)	0.0183 (9)	−0.0033 (7)	0.0027 (7)	0.0020 (7)
C7	0.0150 (9)	0.0154 (9)	0.0173 (9)	−0.0021 (7)	0.0025 (7)	−0.0016 (8)
C8	0.0200 (10)	0.0182 (11)	0.0198 (10)	−0.0057 (8)	−0.0004 (8)	0.0001 (8)
C9	0.0139 (9)	0.0151 (10)	0.0172 (9)	−0.0006 (7)	0.0005 (7)	0.0002 (7)
C10	0.0146 (8)	0.0158 (10)	0.0180 (10)	−0.0010 (8)	−0.0011 (7)	0.0029 (8)
C11	0.0148 (9)	0.0159 (10)	0.0205 (10)	−0.0020 (8)	−0.0015 (8)	0.0057 (8)
C12	0.0198 (9)	0.0130 (9)	0.0166 (9)	−0.0066 (8)	−0.0032 (7)	0.0032 (8)
C13	0.0182 (9)	0.0142 (9)	0.0175 (9)	−0.0053 (8)	−0.0014 (7)	0.0025 (8)
C14	0.0212 (10)	0.0188 (10)	0.0191 (10)	−0.0034 (8)	−0.0042 (8)	0.0031 (8)
C15	0.0205 (10)	0.0158 (10)	0.0317 (12)	−0.0028 (8)	−0.0039 (9)	0.0067 (9)
C16	0.0229 (10)	0.0138 (10)	0.0333 (13)	−0.0026 (8)	0.0058 (9)	−0.0005 (9)
C17	0.0262 (11)	0.0153 (10)	0.0186 (10)	−0.0063 (8)	0.0017 (8)	0.0009 (8)
C18	0.0179 (9)	0.0120 (9)	0.0201 (10)	−0.0062 (7)	−0.0017 (7)	0.0031 (8)
C19	0.0177 (9)	0.0140 (10)	0.0181 (9)	−0.0078 (7)	−0.0021 (8)	0.0030 (8)

### Geometric parameters (Å, °)

**Table:**

O1—C1	1.412 (3)	C4—C5	1.523 (3)
O1—H1	0.81 (3)	C4—H4A	0.9900
O2—C3	1.431 (2)	C4—H4B	0.9900
O2—H2A	0.84 (3)	C5—H5	1.0000
O3—C5	1.425 (2)	C6—C7	1.334 (3)
O3—C2	1.440 (2)	C6—H6	0.9500
O4—C10	1.219 (2)	C7—C9	1.452 (3)
O5—C9	1.219 (2)	C7—C8	1.497 (3)
O6—C19	1.211 (2)	C8—H8A	0.9800
O7—C12	1.202 (2)	C8—H8B	0.9800
N1—C10	1.380 (2)	C8—H8C	0.9800
N1—C6	1.382 (2)	C11—H11A	0.9900
N1—C5	1.471 (3)	C11—H11B	0.9900
N2—C10	1.395 (3)	C12—C13	1.492 (3)
N2—C9	1.407 (2)	C13—C14	1.378 (3)
N2—C11	1.465 (3)	C13—C18	1.388 (3)
N3—C19	1.388 (3)	C14—C15	1.392 (3)
N3—C12	1.409 (3)	C14—H14	0.9500
N3—C11	1.453 (3)	C15—C16	1.387 (3)
C1—C2	1.528 (3)	C15—H15	0.9500
C1—H1A	0.9900	C16—C17	1.398 (3)
C1—H1B	0.9900	C16—H16	0.9500
C2—C3	1.522 (3)	C17—C18	1.375 (3)
C2—H2	1.0000	C17—H17	0.9500
C3—C4	1.517 (3)	C18—C19	1.490 (3)
C3—H3	1.0000		
			
C1—O1—H1	107 (2)	C6—C7—C9	118.41 (17)
C3—O2—H2A	109.2 (19)	C6—C7—C8	123.23 (18)
C5—O3—C2	109.66 (14)	C9—C7—C8	118.32 (17)
C10—N1—C6	122.41 (16)	C7—C8—H8A	109.5
C10—N1—C5	117.88 (16)	C7—C8—H8B	109.5
C6—N1—C5	119.67 (16)	H8A—C8—H8B	109.5
C10—N2—C9	125.79 (16)	C7—C8—H8C	109.5
C10—N2—C11	117.40 (16)	H8A—C8—H8C	109.5
C9—N2—C11	116.80 (16)	H8B—C8—H8C	109.5
C19—N3—C12	112.37 (16)	O5—C9—N2	119.29 (18)
C19—N3—C11	124.13 (16)	O5—C9—C7	125.11 (18)
C12—N3—C11	123.48 (17)	N2—C9—C7	115.60 (17)
O1—C1—C2	113.36 (17)	O4—C10—N1	122.69 (18)
O1—C1—H1A	108.9	O4—C10—N2	123.01 (18)
C2—C1—H1A	108.9	N1—C10—N2	114.30 (16)
O1—C1—H1B	108.9	N3—C11—N2	112.49 (16)
C2—C1—H1B	108.9	N3—C11—H11A	109.1
H1A—C1—H1B	107.7	N2—C11—H11A	109.1
O3—C2—C3	105.44 (15)	N3—C11—H11B	109.1
O3—C2—C1	112.05 (16)	N2—C11—H11B	109.1
C3—C2—C1	111.39 (16)	H11A—C11—H11B	107.8
O3—C2—H2	109.3	O7—C12—N3	125.30 (19)
C3—C2—H2	109.3	O7—C12—C13	129.58 (19)
C1—C2—H2	109.3	N3—C12—C13	105.12 (16)
O2—C3—C4	106.77 (16)	C14—C13—C18	121.50 (19)
O2—C3—C2	110.83 (16)	C14—C13—C12	130.12 (19)
C4—C3—C2	100.89 (15)	C18—C13—C12	108.38 (17)
O2—C3—H3	112.5	C13—C14—C15	117.4 (2)
C4—C3—H3	112.5	C13—C14—H14	121.3
C2—C3—H3	112.5	C15—C14—H14	121.3
C3—C4—C5	101.91 (15)	C16—C15—C14	121.2 (2)
C3—C4—H4A	111.4	C16—C15—H15	119.4
C5—C4—H4A	111.4	C14—C15—H15	119.4
C3—C4—H4B	111.4	C15—C16—C17	121.1 (2)
C5—C4—H4B	111.4	C15—C16—H16	119.5
H4A—C4—H4B	109.3	C17—C16—H16	119.5
O3—C5—N1	108.46 (16)	C18—C17—C16	117.3 (2)
O3—C5—C4	106.03 (15)	C18—C17—H17	121.4
N1—C5—C4	113.97 (16)	C16—C17—H17	121.4
O3—C5—H5	109.4	C17—C18—C13	121.61 (19)
N1—C5—H5	109.4	C17—C18—C19	130.20 (19)
C4—C5—H5	109.4	C13—C18—C19	108.19 (17)
C7—C6—N1	123.40 (18)	O6—C19—N3	124.58 (19)
C7—C6—H6	118.3	O6—C19—C18	129.48 (19)
N1—C6—H6	118.3	N3—C19—C18	105.92 (16)
			
C5—O3—C2—C3	−16.30 (19)	C11—N2—C10—O4	3.0 (3)
C5—O3—C2—C1	105.04 (18)	C9—N2—C10—N1	2.3 (3)
O1—C1—C2—O3	48.5 (2)	C11—N2—C10—N1	−176.37 (17)
O1—C1—C2—C3	166.33 (16)	C19—N3—C11—N2	63.4 (2)
O3—C2—C3—O2	−78.12 (18)	C12—N3—C11—N2	−118.22 (19)
C1—C2—C3—O2	160.12 (16)	C10—N2—C11—N3	−112.99 (19)
O3—C2—C3—C4	34.68 (18)	C9—N2—C11—N3	68.2 (2)
C1—C2—C3—C4	−87.08 (19)	C19—N3—C12—O7	178.40 (19)
O2—C3—C4—C5	76.85 (18)	C11—N3—C12—O7	−0.2 (3)
C2—C3—C4—C5	−39.01 (18)	C19—N3—C12—C13	−1.4 (2)
C2—O3—C5—N1	−131.95 (16)	C11—N3—C12—C13	−179.95 (16)
C2—O3—C5—C4	−9.2 (2)	O7—C12—C13—C14	1.6 (4)
C10—N1—C5—O3	−135.93 (17)	N3—C12—C13—C14	−178.7 (2)
C6—N1—C5—O3	41.6 (2)	O7—C12—C13—C18	−178.3 (2)
C10—N1—C5—C4	106.2 (2)	N3—C12—C13—C18	1.5 (2)
C6—N1—C5—C4	−76.3 (2)	C18—C13—C14—C15	−0.6 (3)
C3—C4—C5—O3	30.72 (18)	C12—C13—C14—C15	179.57 (19)
C3—C4—C5—N1	149.95 (16)	C13—C14—C15—C16	0.2 (3)
C10—N1—C6—C7	−1.3 (3)	C14—C15—C16—C17	0.9 (3)
C5—N1—C6—C7	−178.70 (19)	C15—C16—C17—C18	−1.5 (3)
N1—C6—C7—C9	−0.1 (3)	C16—C17—C18—C13	1.1 (3)
N1—C6—C7—C8	177.74 (18)	C16—C17—C18—C19	−177.88 (19)
C10—N2—C9—O5	176.57 (19)	C14—C13—C18—C17	−0.1 (3)
C11—N2—C9—O5	−4.8 (3)	C12—C13—C18—C17	179.79 (18)
C10—N2—C9—C7	−3.6 (3)	C14—C13—C18—C19	179.10 (18)
C11—N2—C9—C7	175.06 (17)	C12—C13—C18—C19	−1.0 (2)
C6—C7—C9—O5	−177.8 (2)	C12—N3—C19—O6	179.60 (19)
C8—C7—C9—O5	4.2 (3)	C11—N3—C19—O6	−1.9 (3)
C6—C7—C9—N2	2.4 (3)	C12—N3—C19—C18	0.8 (2)
C8—C7—C9—N2	−175.61 (17)	C11—N3—C19—C18	179.35 (17)
C6—N1—C10—O4	−179.05 (19)	C17—C18—C19—O6	0.6 (4)
C5—N1—C10—O4	−1.6 (3)	C13—C18—C19—O6	−178.5 (2)
C6—N1—C10—N2	0.3 (3)	C17—C18—C19—N3	179.28 (19)
C5—N1—C10—N2	177.71 (17)	C13—C18—C19—N3	0.2 (2)
C9—N2—C10—O4	−178.40 (19)		

### Hydrogen-bond geometry (Å, °)

**Table:**

*D*—H···*A*	*D*—H	H···*A*	*D*···*A*	*D*—H···*A*
O1—H1···O2^i^	0.81 (3)	2.02 (3)	2.815 (2)	170 (3)
O2—H2A···O6^i^	0.84 (3)	1.92 (3)	2.698 (2)	155 (3)
C3—H3···O6^ii^	1.00	2.45	3.445 (2)	171
C8—H8A···O7^iii^	0.98	2.43	3.341 (3)	154
C14—H14···O5^iv^	0.95	2.44	3.116 (3)	128
C15—H15···O7^iv^	0.95	2.60	3.484 (3)	155
C5—H5···O4	1.00	2.33	2.740 (2)	104
C11—H11A···O4	0.99	2.29	2.747 (2)	107
C11—H11B···O7	0.99	2.52	2.915 (3)	104
C8—H8B···Cg1^v^	0.98	2.71	3.534 (2)	143
C11—H11A···Cg2^vi^	0.99	2.73	3.611 (2)	149

Symmetry codes: (i) −*x*+2, *y*−1/2, −*z*+2; (ii) −*x*+1, *y*−1/2, −*z*+2; (iii) −*x*+1, *y*−1/2, −*z*+1; (iv) −*x*, *y*+1/2, −*z*+1; (v) *x*−1, *y*, *z*; (vi) *x*+1, *y*, *z*.
